# Genetic targeting of *NRXN2* in mice unveils role in excitatory cortical synapse function and social behaviors

**DOI:** 10.3389/fnsyn.2015.00003

**Published:** 2015-02-19

**Authors:** Gesche Born, Hannah M. Grayton, Hanna Langhorst, Irina Dudanova, Astrid Rohlmann, Benjamin W. Woodward, David A. Collier, Cathy Fernandes, Markus Missler

**Affiliations:** ^1^Institute of Anatomy and Molecular Neurobiology, Westfälische Wilhelms-UniversityMünster, Germany; ^2^Social, Genetic and Developmental Psychiatry Centre, Institute of Psychiatry, Psychology and Neuroscience, King's College LondonLondon, UK; ^3^Discovery Neuroscience Research, Eli Lilly and Company Ltd.Surrey, UK; ^4^Cluster of Excellence EXC 1003, Cells in MotionMünster, Germany

**Keywords:** vesicle release, synaptic plasticity, cognition, electron microscopy, AMPA receptor, NMDA receptor, anxiety-like behavior, social interaction

## Abstract

Human genetics has identified rare copy number variations and deleterious mutations for all neurexin genes (*NRXN1-3*) in patients with neurodevelopmental diseases, and electrophysiological recordings in animal brains have shown that Nrxns are important for synaptic transmission. While several mouse models for Nrxn1α inactivation have previously been studied for behavioral changes, very little information is available for other variants. Here, we validate that mice lacking Nrxn2α exhibit behavioral abnormalities, characterized by social interaction deficits and increased anxiety-like behavior, which partially overlap, partially differ from Nrxn1α mutant behaviors. Using patch-clamp recordings in Nrxn2α knockout brains, we observe reduced spontaneous transmitter release at excitatory synapses in the neocortex. We also analyse at this cellular level a novel *NRXN2* mouse model that carries a combined deletion of Nrxn2α and Nrxn2β. Electrophysiological analysis of this Nrxn2-mutant mouse shows surprisingly similar defects of excitatory release to Nrxn2α, indicating that the β-variant of Nrxn2 has no strong function in basic transmission at these synapses. Inhibitory transmission as well as synapse densities and ultrastructure remain unchanged in the neocortex of both models. Furthermore, at Nrxn2α and Nrxn2-mutant excitatory synapses we find an altered facilitation and *N*-methyl-D-aspartate receptor (NMDAR) function because NMDAR-dependent decay time and NMDAR-mediated responses are reduced. As Nrxn can indirectly be linked to NMDAR via neuroligin and PSD-95, the trans-synaptic nature of this complex may help to explain occurrence of presynaptic and postsynaptic effects. Since excitatory/inhibitory imbalances and impairment of NMDAR function are alledged to have a role in autism and schizophrenia, our results support the idea of a related pathomechanism in these disorders.

## Introduction

Synapse function and plasticity in the brain depend on the coordinated action of molecular machineries across the synaptic cleft. Presynaptic neurexins (Nrxns) form *trans*-synaptic complexes with postsynaptic neuroligins and other partners that are suited to mediate such function (Missler et al., 2012; Reissner et al., 2013). Consistent with this role, an association has been established between copy number variations or hemizygous exonic deletions within the NRXN1 gene (2p16.3) and neurodevelopmental disorders, including autism, schizophrenia and intellectual disability (Feng et al., 2006; Szatmari et al., 2007; Kim et al., 2008; Kirov et al., 2008; Yan et al., 2008; Zahir et al., 2008; Rujescu et al., 2009; Zweier et al., 2009; Ching et al., 2010; Voineskos et al., 2011; Yue et al., 2011; Buxbaum et al., 2012; Camacho-Garcia et al., 2012; Liu et al., 2012; Hoeffding et al., 2014).

Vertebrates contain three NRXN genes (NRXN1-3) that each produce longer α-Nrxn and shorter β-Nrxn extracellular isoforms, from independent promoters (Tabuchi and Sudhof, 2002; Runkel et al., 2013). These are then alternatively spliced at five, possibly six, conserved sites (Tabuchi and Sudhof, 2002; Treutlein et al., 2014). α-Nrxns encode six LNS domains with three interspersed EGF-like domains, while β-Nrxns contain a single LNS-domain that is identical to the sixth LNS-domain of the corresponding α-Nrxn, and mediates binding, for example, to neuroligins (Ichtchenko et al., 1995; Boucard et al., 2005; Reissner et al., 2008). Studies of knockout mice have revealed essential functions for α-Nrxns and neuroligins at excitatory and inhibitory synapses (Missler et al., 2003; Varoqueaux et al., 2006). Multiple knockout of all three α-Nrxns is lethal at birth due to a dramatic impairment of Ca^2+^-dependent vesicle release (Missler et al., 2003; Kattenstroth et al., 2004; Zhang et al., 2005), but even removal of a single Nrxn1α isoform reduces glutamatergic transmission (Missler et al., 2003; Etherton et al., 2009). While levels of β-Nrxns appeared unchanged in α-Nrxns KO mice (Missler et al., 2003), no information of deletion of β-Nrxns in mice is available yet.

In studies on autism spectrum disorders, which are characterized clinically by impairments in reciprocal social interactions and communication as well as stereotyped behaviors, rare heterozygous deletions in the NRXN1 gene are considered as causative variants (Delorme et al., 2013). Consequently, several studies have addressed higher brain functions in NRXN1 mouse models by behavioral profiling (Ey et al., 2011). Nrxn1α homozygote KO mice display a decrease in social investigation and an increase in aggressive behavior as well as reduced locomotor activity in novel environments (Grayton et al., 2013), impairments in prepulse inhibition of the startle response, deficits in nest building activities, and an improvement in motor learning (Etherton et al., 2009). Furthermore, Nrxn1α heterozygote mice have been shown to have sex-dependent increases in response to novelty and accelerated habituation to novel environments (Laarakker et al., 2012).

Although genetic studies also revealed disease-associated mutations in NRXN2 (11q13) and NRXN3 (14q31) genes (Gauthier et al., 2011; Vaags et al., 2012), only limited data are available on changes in behavior from mouse models (Dachtler et al., 2014). Here, we assessed behaviors in Nrxn2α KO mice and performed electrophysiological recordings from neocortical neurons. Behavioral testing confirmed impairments of social interaction and anxiety-like behaviors. Since deletions in humans not only affect the 5′-exons that encode for the α-Nrxn isoform but may extend into exons encoding β-Nrxn (Feng et al., 2006; Ching et al., 2010) we asked whether the simultaneous deletion of α- and β-variants of the NRXN2 gene would produce a cellular phenotype that is more severe than Nrxn2α mutations alone. *In-situ* hybridization data for Nrxn2 variants have shown that Nrxn2α expression is present in the neocortex and differentially enhanced in layers 2, 4, and 6, whereas Nrxn2β is more evenly expressed. Outside the cortex, Nrxn2α signals are stronger in the septal nuclei, the reticular thalamic nucleus, and some midbrain nuclei, and Nrxn2β in septal nuclei and cerebellum (Ullrich et al., 1995). In our current study, comparison of the single Nrxn2α KO (Missler et al., 2003) and a combined Nrxn2αβ KO by electrophysiology demonstrates a surprisingly similar impairment of excitatory synaptic transmission and NMDAR function. Our data suggest that behaviors and signaling pathways relevant to human disease ethiology are affected in these mouse models.

## Materials and methods

### Animal models

#### Nrxn2α mouse model

Nrxn2α KO mice were generated previously (Missler et al., 2003). For the behavioral study, we subjected the line to 8 generations of additional backcrossing to transfer the knockout allele onto a C57BL/6J genetic background. From the offspring of the F8 pairing, heterozygous mice were crossed together to generate the test mice, including wildtype (WT), heterozygote knockout (HET), and homozygote knockout (KO) mice (*n* = 8–15 per genotype, per sex). All mice were individually housed 1 week prior to behavioral testing with *ad libitum* access to water and food. All procedures were performed in compliance with the local ethical review panel of King's College, and U.K. Home Office Animals Scientific Procedures Act 1986 (license PPL:70/7184). Other experiments were approved by the Landesamt für Natur, Umwelt und Verbraucherschutz NRW, Germany (license 84- 02.05.20.11.209).

#### Combined Nrxn2 mouse model

To delete the α- and β-variants of the Nrxn2 gene together, part of exon 23 (Tabuchi and Sudhof, 2002) was deleted, and the gene interrupted between splice insert #5 into the 3′UTR. Mutant mice were originally generated in Thomas C. Südhof's laboratory (Stanford University, USA), which will also provide additional analyses at a later time point. Genotyping was performed by PCR with oligonucleotide primer combinations: 5′-AGC CCC GAC CCA ACC TCA GGA CAG A -3′ vs. 5′-GGT AGG GAC AAG AGA CAG CAA-3′ (WT allele, 1 000 bp product), and 5′-CGC CGC TCC CGA TTC GCA GCG CAT-3′ vs. 5′-GGT AGG GAC AAG AGA CAG CAA-3′ (KO allele, 700 bp product).

### Behavioral testing (Nrxn2α mice)

WT, HET, and KO mice were 10 weeks old at testing and recorded using a camera above test arenas. Movement of each mouse was tracked using EthoVision software (Noldus Information Technologies, Wageningen, Netherlands). All mice underwent each task and were assessed in the following order: home cage, open field, light/dark box, elevated plus maze, three-chamber social approach task, nesting, and grooming behavior, social investigation with a juvenile conspecific, Morris water maze, delayed matching-to-place task in the water maze, and food burying test for olfactory ability. There was at least 1 day rest allowed between tasks.

The behavioral battery applied was recently described in our analysis of Nrxn1α mutants (Grayton et al., 2013). Briefly, to probe *spontaneous locomotor activity:* Mice were monitored in a homecage and locomotor activity was recorded at three 1 h periods (namely 12pm, 1am, and 11am the following morning). *Anxiety tasks:* open field, light/dark box, and elevated plus maze were performed. For the open field task, animals were placed in the arena for 10 min and their behavior was monitored across three areas defined by EthoVision software, namely the outer, central, and inner zones. The light/dark box trial was 5 min in duration, and the animals were free to explore both the smaller darkly lit chamber (20 lux) and the larger brightly illuminated chamber (80–110 lux). Lastly, during the elevated plus maze task mice were placed on the elevated central platform and left to explore the open and closed arms of the maze for 5 min. For all three tasks activity was measured in the least anxiogenic part of the arenas, namely the outer zone of the open field, the dark compartment of the light/dark box and the closed arms of the elevated plus maze, For anxiety, the time spent in the most anxiogenic part of the arenas were measured, namely the central zone of the open field, the light compartment of the light/dark box and the open arms of the elevated plus maze. *Cognitive tasks:* a range of cognitive tasks were carried out, including Morris water maze (MWM) and delayed matching-to-place (DMP). MWM was performed with mice run in squads of 6 mice/squad. Each mouse underwent four trials per day. Mice were tested for 10 consecutive days and a probe task was run on the last day to assess the retention of spatial memory. DMP was also carried out using the MWM - animals underwent 8 trials/day for 7 days, the platform location was changed each day in a random manner, and the maximum trial length was 90 s. Reduction in latencies to find the platform between the first and subsequent trials is referred to as “saving time” and is used as an index of working/episodic-like memory. *Social tasks:* three-chamber social approach task and the social investigation task were both performed. For the three-chamber social approach task, the Ethovision tracking system was used to monitor mouse movements throughout the three chambers over three 10 min trials. During trial 1, the apparatus was empty. In trial 2, one wire cup was placed upside down in one of the side chambers and a novel juvenile conspecific mouse was placed under another wire cup in the other side chamber, leaving the middle chamber empty. The location of the novel mouse across trials was counterbalanced. In trial 3, a novel juvenile conspecific mouse was placed under the second, empty wire cup with the now familiar mouse remaining under the other wire cup. The three trials run successively for each mouse, with an inter-trial delay of 2–3 min as the objects, and conspecific mice were moved. For the social investigation task, if prolonged periods of aggression was seen throughout the 4 min trial (>45 s), trial was stopped and the conspecific mouse was removed. *Grooming behaviors:* Grooming was investigated during a 10 min trial, and recorded by an investigator blind to the genotype. Immediately before the trial the mice were placed in a clean, standard homecage, with no sawdust or nesting material, and allowed to habituate for 10 min under red light. *Nesting behaviors:* on day 1, mice were placed in a fresh home cage with 60 g of standard food and 90 g of sawdust. 20 g of nesting material was placed in the food hopper on top of the cage. The amount of nesting material left on the food hopper and pulled into the cage was measured 24 h later. In addition, the dimensions (cm) and weight (g) of the nest were measured. *Buried food task:* this was performed as described (Grayton et al., 2013), with small chocolate cookies (Nestle Cookie Crisp®, Welwyn Garden City, U.K.) used as the palatable food.

### Electrophysiological recordings (Nrxn2α and Nrxn2 mice)

Mutant mice from both models and littermate controls were used for whole-cell patch-clamp recordings at P14-P21 as decribed (Born et al., 2014). Briefly, animals were anesthetized with isoflurane and brains transferred into ice-cold artificial cerbro-spinal fluid (ACSF; in mM: 118 NaCl, 3 KCl, 1 NaH_2_PO_4_, 20 glucose, 1.5 CaCl_2_, 1 MgCl_2_, 25 NaHCO_3_, pH 7.3, ≈305 mosmol), continuously aerated with 95% O_2_ and 5% CO_2_. Recordings were performed on layer V pyramidal cells of the primary somatosensory cortex in acute frontal slices (400 μm). Pipettes made of borosilicate glass tubes were filled with internal solution (in mM: 140 Kaliumgluconate, 1 CaCl_2_, 10 HEPES, 2 MgCl_2_, 4 Na-ATP, 0.5 Na-GTP, 10 EGTA, pH 7.3, 300 mosmol), supplemented with 5 mM lidocain (QX-314) to prevent generation of sodium spikes. Passive membrane properties were documented, and cells with membrane resistances between 80 and 800 MΩ, resting potentials between −55 and −80 mV, and a capacity of 25–150 pF were selected for further analysis. Excitatory miniature postsynaptic currents (mEPSCs) were measured under 1 μM TTX (Tocris) and 10 μM bicuculline (Sigma Aldrich) at −80 mV, and mIPSCs under TTX and additional application of the AMPA- and Kainate receptor antagonist CNQX (20 μM, Sigma Aldrich) and the selective NMDA receptor antagonist APV (25 μM, Sigma Aldrich) at −30 mV. Signals that were at least three times larger than background noise were selected for statistical analysis.

Electrically evoked EPSCs (eEPSCs) were recorded at −80 mV near the calculated reversal potential of chloride (−65 mV), with no additional pharmacology. Evoked IPSCs were recorded in presence of 25 μM APV and 20 μM CNQX at holding potentials of −30 mV. Single pulses (duration: 90 μs) were induced with a concentric bipolar stimulation electrode (SNEX-100X) that was placed approximately 100 μm laterally to the recording position at increasing stimulation strengths. For paired-pulse experiments at half maximum strength (values within the range of 80–100 μA), stimuli were applied 10× at intervals of 20 s to avoid summation. The paired-pulse ratio (PPR) is defined by the ratio of the second current (amplitude) to the first one. Inter-stimulus intervals varied from 20 to 200 ms (20, 30, 50, 75, 100, 125, 150, 175, 200 ms respectively). In addition, stimulus trains with ten pulses of 20 Hz were used to induce short-term plasticity. Each train was recorded five times with an interval of 20 s. In some of the evoked EPSC experiments, the NMDA receptor blocker APV was applied to the bath medium (25 μM), or MK-801 (1 mM) was added to the internal pipette solution. Analysis of the 20 Hz stimulus train was defined by the ratios of the second to the first, the fifth to the first, and the tenth to the first amplitude for each experiment. To determine the NMDA/AMPA ratio (Myme et al., 2003), mixed NMDA/AMPA EPSCs were evoked using single electrical stimulation, and recordings were performed 10 times with an inter-stimulus interval of 15 s. EPSCs were recorded from −80 mV in intermediate steps of 20 mV to +40 mV. To estimate the AMPA receptor-mediated current at +40 mV the time-point of the peak at −80 mV was determined (10 ms). The NMDAR-mediated component of the EPSC was choosen 40 ms after the stimulus, and NMDA/AMPA ratio was determined. All signals were digitizied and amplified using an EPC-10 USB amplifier (HEKA Elektronik, Germany). Currents were filtered by a bessel filter at a frequency of 2.9 kHz and digitized at a sampling rate of 20 kHz. Data acquisition and analysis were performed using commercially available software from HEKA (PatchMaster V2X42 and FitMaster V2X32, HEKA Elektronik, Germany), MiniAnalysis (Synaptosoft, Decatur, GA) and Microsoft Excel.

### Electron microscopy (Nrxn2α and Nrxn2 mice)

Anesthetized mice were transcardially perfused with 70 ml of 2% glutaraldehyde (Serva, Heidelberg, Germany) and 2% paraformaldehyde (Merck, Darmstadt, Germany) in 0.1 M PB at 37°C, and postfixed at 4°C overnight. Blocks of cortical tissue were contrasted in 1% OsO_4_ for 2 h at RT. Following washes with dH_2_O and dehydrating, tissue was incubated with propylene oxide (Electron Microscopy Science, EMS, Hatfield, USA) for 45 min, infiltrated with propylene oxide/epon (1:1) for 1 h, in pure epon resin (EMS) overnight, and hardened at 60°C for 24 h. Contrasting of thin sections was done on Formvar-coated copper grids with a saturated solution of 12% uranyl acetate and lead citrate. Samples were investigated with a transmission electron microscope (Libra 120, Zeiss) at 80 kV, and images taken with a 2048 × 2048 CCD camera (Tröndle, Moorenweis, Germany). Two image series from the somatosensory cortex of each animal were examined at 8000X primary magnification. Each series included all cortical layers, and represented an area of about 1500 μm^2^. Asymmetric type 1 synapses were defined as contacts with a visible synaptic cleft, a distinct postsynaptic density (PSD) and at least three synaptic vesicles, whereas symmetric (type 2) contacts showed an apparent PSD and contained pleiomorphic vesicles (Gray, 1959), and both populations were quantified as area densities. In addition, randomly chosen asymmetric synapses were analyzed at a higher zoom level to quantify the presynaptic terminal area, density of synaptic vesicles per terminal area, width of synaptic cleft, and length of active zone.

### RNA work

Total RNA from mouse whole brains (brain stem cut away) at different developmental stages (P1, P4, P7, P10, P14, P20 and P50–60) was isolated immediately after dissection with RNAzolB (WAK Chemie, Germany). Northern blotting experiments were performed with total RNA on formaldehyde gels as described (Beglopoulos et al., 2005), using two different ^32^P-radiolabeled probes: 5′-probe, 900 bp-long *Nco*I/*BamH*I fragment (bp 70–970) cut from cDNA plasmid p520-1a; 3′-probe, 530 bp-long *Sfi*I/*Kpn*I fragment (bp 4220–4900) cut from plasmid pCMV-L1. All blots were re-hybridized with a β −actin probe to control for RNA loading.

For PCR, RNA was reverse-transcribed with GeneAmp Gold RNA PCR Core Kit using Oligo dT primer. Real-time PCR was performed with isoform-specific primers and SYBR Green PCR Master Mix in an ABI Prism 7000 Sequence Detection System, using oligonucleotide primers: for Nrxn2α, 5′-CTACCTTCTGCTG GACATGGGCTCC-3′ (in exon 8) vs. 5′-GCGTGCTGCGGCTGTTCACA-3′ (in exon 9), generating a 140 bp product; for Nrxn2β, 5′-GTCTCGTCCAGCCTCAGCACCACC-3′ (located in β-specific exon) vs. 5′-CGTGTACTGGGCCGGTCATTGGGA-3′ (in exon 18), generating a 188 bp product. All reactions were performed in duplicates with β-actin as reference. Signals were analyzed by ABI Prism Sequence Detection Software (Applied Biosystems), and the ΔΔC_*t*_ method was used for relative quantification of Nrxn2 transcripts. Standard curves were generated with serial five-fold dilutions of cDNA in triplicates, and efficiency of amplification was calculated from the slope of the standard curve as *E* = 10^−1/*k*^, where E is amplification efficiency and k is the slope. The efficiency was between 79 and 100% and was taken into consideration when calculating the relative expression levels. DNA melting curves were generated after each experiment to confirm the specificity of amplification.

### Statistical analysis

Data presented are means ± SEM. Statistical significance was tested with a two-tailed unpaired Student's *t*-test using Prism 4.0 a software (GraphPad Software, San Diego, USA), assuming Gaussian distribution. Results were denoted statistically significant when *P*-values were <0.05 (significance levels as indicated in Figure legends; number (*n*) of samples/repeats are given in the Results and Figure legends.

Behavioral data were analyzed with Statistica software (Version 5.5, StatSoft, Inc., Tulsa, OK), using either a Student's *t*-test, a Two-Way ANOVA or a Two-way repeated measures ANOVA, as appropriate. The between-factors were always sex and genotype, and within-factors were either time (home cage), chamber (automated three-chamber social approach task) or sessions (MWM, DMP). Tukey HSD *post-hoc* pairwise comparisons were performed on significant results. An analysis of covariance (ANCOVA) was used to look at the relationship between activity and anxiety measures.

## Results

### Behavioral testing of Nrxn2α KO mice

#### Increased anxiety-like behaviors

**Figure 1:**
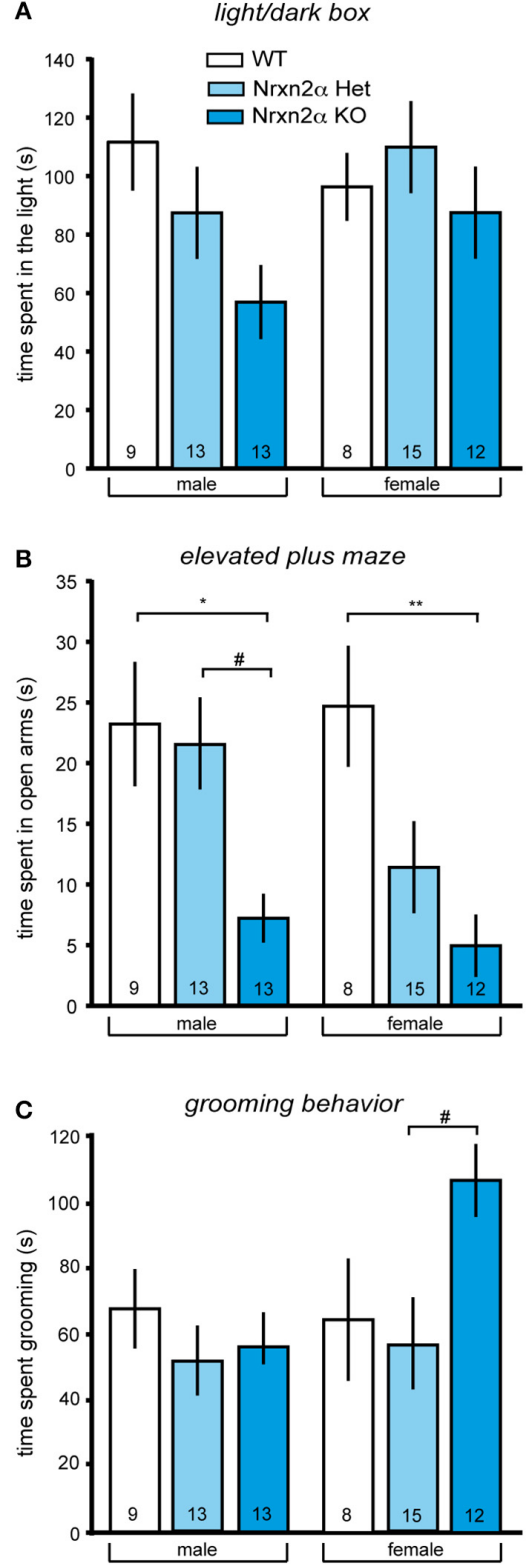
**Increased anxiety-like and repetitive behaviors in Nrxn2α-deficient mice**. Anxiety behavior of Nrxn2α KO was assessed in the light/dark box and the elevated plus maze. **(A)** Time (s) spent in the light compartment of the light/dark box male (left pair of bars) and female mice (right pair of bars), respectively. **(B)** Time (s) spent in the open arms of the elevated plus maze by male (left pair of bars) and female (right pair of bars) mice, respectively. **(C)** Time (s) spent in grooming was quantitated to analyze repetitive behaviors of male (left pair of bars) and female mice (right pair of bars). Data shown are mean (± SEM) and derived from 17 WT (white bars, 9♂, 8♀), 28 HET (light blue bars, 13♂, 15♀), and 25 Nrxn2α KO mice (blue squares, 13♂, 12♀); level of significance indicated by ^*^p < 0.05 and ^**^p < 0.01, compared to WT and #*p* < 0.05, compared to HET.

**Figure 2:**
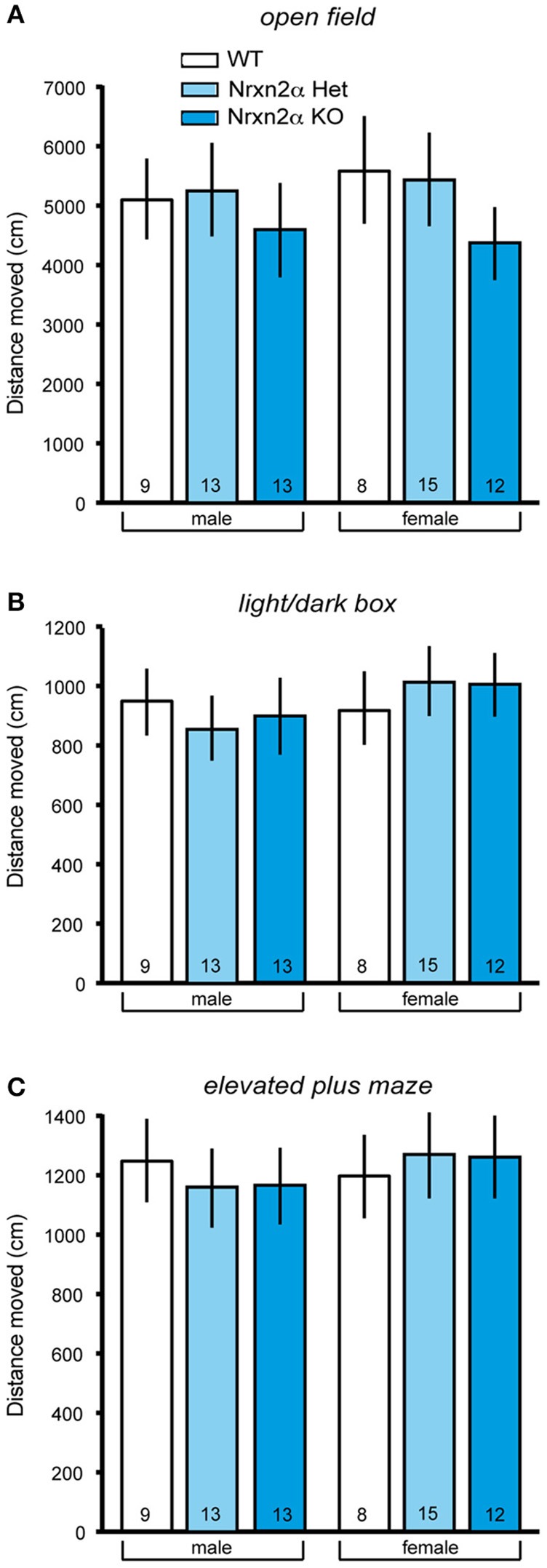
**Locomotor activity measures of Nrxn2α-deficient mice**. Locomotor activity was measured in the open field, light/dark box and elevated plus maze. **(A)** Distance (cm) traveled in the outer zone of the open field (left pair of bars) and female mice (right pair of bars), respectively. **(B)** Distance (cm) traveled in the dark compartment of the light/dark box (left pair of bars) and female mice (right pair of bars), respectively. **(C)** Distance (cm) traveled in the closed arms of the elevated plus maze (left pair of bars) and female mice (right pair of bars), respectively. Data shown are mean (± SEM) and derived from 17 WT (white bars, 9♂, 8♀), 28 HET (light blue bars, 13♂, 15♀), and 25 Nrxn2α KO mice (blue squares, 13♂, 12♀).

To ensure comparability with the analysis of Nrxn1α KOs (Grayton et al., 2013), we first studied anxiety-like behaviors (Figure 1) and locomotor activity (Figure 2) in Nrxn2α KO mice backcrossed to a single C57BL/6J background. Spontaneous locomotion was assessed in the homecage at three time points across a 24 h time period. During transfer hour, all mice significantly reduced locomotor activity, suggesting that they had become habituated [sessions factor—*F*_(5, 230)_ = 72.85, *p* < 0.001]. There were no differences in distance traveled between genotype groups in the transfer or light hours. Across the dark hour, there was a significant genotype effect [genotype factor—*F*_(2, 41)_ = 7.14, *p* = 0.002] due to reduced activity by female KO mice. As Nrxn1α mutants displayed reduced locomotor activity in novel environments (Grayton et al., 2013), the activity of Nrxn2α KO was also measured in 3 novel arenas, namely the open field, light/dark box and elevated plus maze. Activity was measured in the least anxiogenic areas (outer area of the open field, dark area of light/dark box and closed arms of elevated plus maze), as here the activity measure is less confounded by anxiety, providing a cleaner measure of locomotor activity (Fernandes and File, 1996). A significant reduction in locomotor activity was seen in the open field as KO mice traveled less in the outer area [Figure 2A; genotype factor: *F*_(2, 64)_ = 6.15, *p* = 0.004]. No activity differences were seen in either the distance traveled in the dark compartment of the light/dark box or in closed arms of the elevated plus maze (Figures 2B,C). Nrxn2α KO mice also exhibited a reduction in speed in the outer area of the open field [genotype factor: *F*_(2, 64)_ = 8.83, *p* < 0.001] and the closed arms of the maze [genotype factor: *F*_(2, 64)_ = 4.33, *p* = 0.017].

In addition to locomotor activity, anxiety can be measured in these tasks, using time spent in the central zone of the open field, light compartment of the light/dark box and open arms of the elevated plus maze. Nrxn2α KO mice spent a shorter amount of time in anxiogenic light of the light/dark box [Figure 1A; genotype factor—*F*_(2, 64)_ = 3.18, *p* = 0.048; sex factor—*F*_(1, 64)_ = 1.4, *p* = 0.2], and this effect was not being driven by either sex (Tukey HSD; *p* > 0.05). Nrxn2α KO mice also spent a shorter amount of time on the open arms of the elevated plus maze [Figure 1B; genotype factor—*F*_(2, 64)_ = 12.21, *p* < 0.001; sex factor—*F*_(1, 64)_ = 1.7, *p* = 0.2], where significant reductions were seen in both the male and female KO mice (Tukey HSD; *p* < 0.05). For the elevated plus maze, there also a significant genotype effect of time spent on the central platform, as the KO mice spent a significant shorter amount of time here [genotype factor—F_(2, 64)_ = 4.51, *p* = 0.01]. There was no effect of genotype on the time spent in the center area of the open field. ANCOVA was performed for activity and anxiety measures to assess whether activity differences were influencing anxiety. For the light/dark box, the ANCOVA revealed a significant interaction effect [*F*_(1, 63)_ = 20.7, *p* < 0.001], a result which just lost significance when co-varying out locomotor activity [*F*_(2, 63)_ = 2.74, *p* = 0.07]. For elevated plus maze, it produced a significant interaction effect [*F*_(1, 63)_ = 5.19, *p* = 0.026] that remained significant when co-varying out locomotor activity [*F*_(2, 63)_ = 12.11, *p* < 0.001]. These ANCOVA results suggest that an increase in anxiety is likely the main behavior driving the Nrxn2α KO phenotype assessed.

#### Altered communication and social behaviors

As repetitive/stereotyped behaviors are a core feature of autism, grooming behaviors were also measured in Nrxn2α mice. Female but not male KO mice spent a longer time grooming compared to heterozygote mice [Figure 1C; genotype effect—*F*_(2, 64)_ = 3.27, *p* = 0.04; Tukey HSD; *p* = 0.03 for female KO compared to female HET mice]. There were no differences in frequency of grooming bouts between the genotype groups but a significant sex effect for the time spent rearing, as females tended to rear less than males [sex effect—*F*_(1, 64)_ = 7.24, *p* = 0.008].

**Figure 3:**
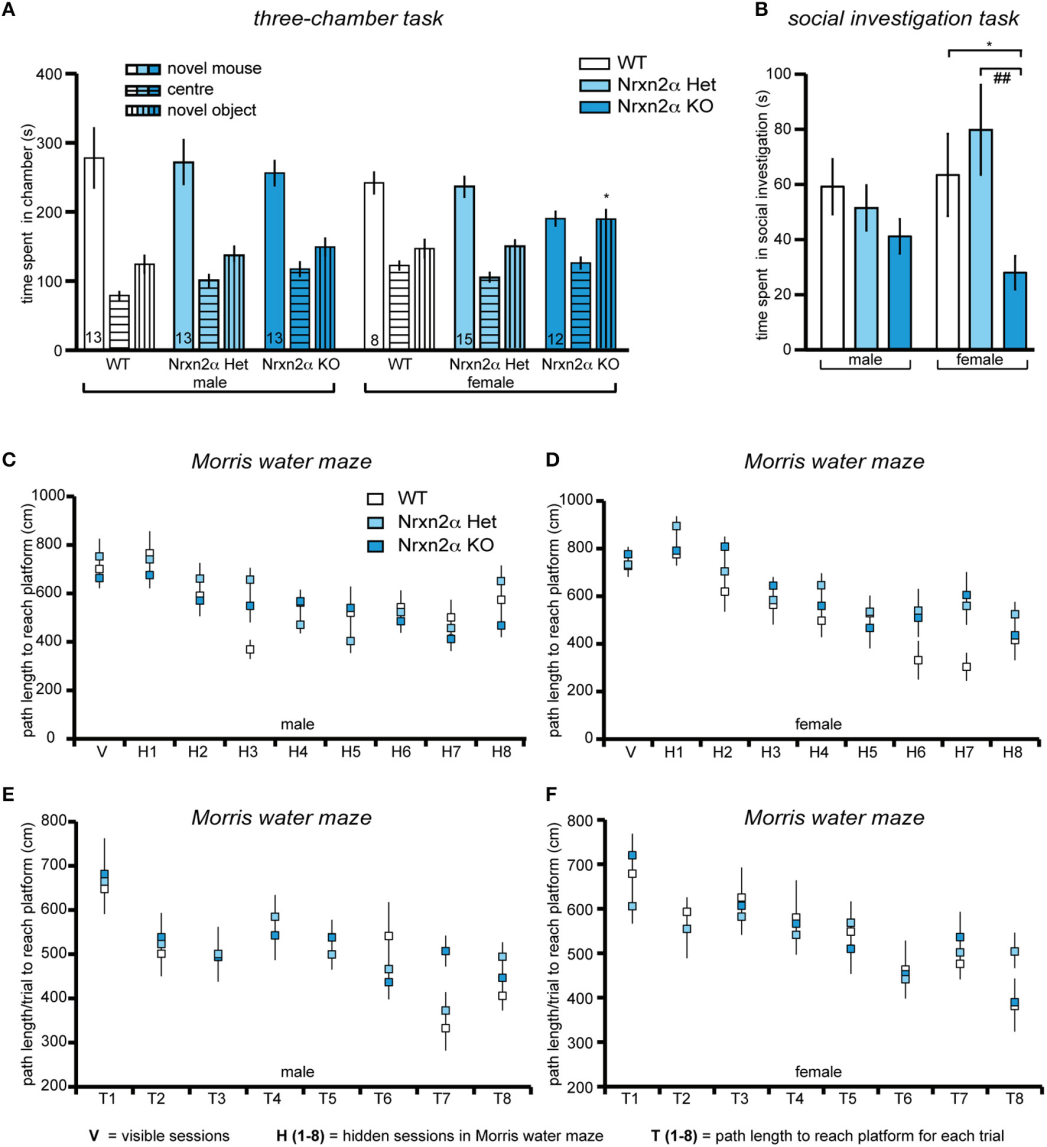
**Deletion of Nrxn2α in mice impaires social interaction behaviors**. Behavioral analysis of Nrxn2α KO mice in the three-chamber social approach task **(A)**, social investigation task **(B)**, Morris water maze **(C,D)** and delayed matching-to-place task **(E,F)**. **(A)** Time (s) spent in the chambers containing the novel object or the conspecific mouse, and the center chamber during trial 2 of the three-chamber social approach task. **(B)** Time (s) spent socially investigating the conspecific during the social investigation task. **(C, D)** Path length (cm) taken to reach the platform across the visible (V) and hidden (H1 to H9) sessions in the Morris water maze, for male and female mice respectively. **(E, F)** Path length (cm) taken to reach the platform for each trial (T1–T8) averaged across each of the seven sessions, during the delayed matching-to-place task, for male and female mice respectively. Data shown are mean (± SEM) and derived from 17 WT (white bars, 9♂, 8♀), 28 HET (light blue bars, 13♂, 15♀), and 25 Nrxn2α KO mice (blue squares, 13♂, 12♀); level of significance indicated by ^*^p < 0.05, compared to WT and ##*p* < 0.01, compared to HET.

Social approach behaviors were investigated using the three-chamber social approach task with three trials that access preference for social cue vs. novel object and preference for social novelty. During trial 1, there was a significant genotype and sex effect for distance traveled in the whole arena [genotype factor—*F*_(2, 64)_ = 3.74, *p* = 0.03; sex factor—*F*_(1, 64)_ = 7.01, *p* = 0.01], driven by a significant reduction in locomotor activity in male KO mice. This effect, however, was not seen in either trial 2 [genotype factor—*F*_(2, 63)_ = 0.23, *p* = 0.8] or trial 3 [genotype factor—*F*_(2, 63)_ = 2.32, *p* = 0.11]. There was also a reduction in speed in the whole arena of male and female KO [genotype factor—*F*_(2, 64)_ = 3.53, *p* = 0.04]. In trial 2, preference for a social cue was indicated by a test mouse spending more time in the chamber containing a novel juvenile conspecific mouse vs. a novel object (Figure 3A). Preference for social cue (sociability) was seen in all male mice and female WT and HET mice [chamber factor—*F*_(1, 63)_ = 69.5, *p* < 0.001]. There were significant chamber x genotype [*F*_(2, 63)_ = 3.68, *p* = 0.03], and chamber x sex effects [F_(1, 63)_ = 10.89, *p* = 0.001], as female KO did not show a preference for social cue. Female Nrxn2α KO also spent less time with the novel conspecific mouse [*t*_(18)_ = 2.71, *p* = 0.01, *d* = 1.28]. The last trial assessed preference for social novelty. Male mice spent significantly longer with the novel conspecific mouse, compared to the familiar conspecific, suggesting preference for social novelty [chamber factor—*F*_(1, 63)_ = 9.36, *p* = 0.003]. There was also a significant sex effect [*F*_(1, 63)_ = 13.7, *p* < 0.001], as none of the female mice showed this preference and all spent roughly equal times with both conspecific mice. In contrast, no effect of genotype on preference for social novelty was observed.

To further investigate the social behaviors of Nrxn2α mice, investigation of an unfamiliar juvenile same-sex conspecific mouse was measured (Figure 3B). Female KO mice spent less time engaging in social sniffing compared to WT and HET mice [*F*_(2, 64)_ = 5.94, *p* = 0.004]. There were no significant effects of genotype on the time spent in anogenital sniffing or aggression, however, there was a significant sex effect for aggression [*F*_(1, 64)_ = 10.91, *p* = 0.001] as no female mice displayed aggressive behavior toward conspecific mice. Since impaired olfaction would compromise a wide range of behaviors, including social interactions, olfaction was assessed in a simple food burying task. There was no significant effect of genotype on the time taken to find the food [genotype factor: *F*_(2, 64)_ = 0.12, *p* = 0.87]. Additionally, we probed nest building behavior. Nrxn2α KO mice had significantly more bedding left in the food hopper at the end of the trial [genotype factor—*F*_(2, 64)_ = 25.6, *p* = 0.000001], and their nests weighed less [genotype factor—*F*_(2, 64)_ = 15.3, *p* < 0.001] and had reduced height [genotype factor—*F*_(2, 64)_ = 21.9, *p* < 0.001]. There were also sex effects for all measures [bedding in hopper—*F*_(1, 64)_ = 23.9, *p* < 0.001; nest weight—*F*_(1, 64)_ = 32.8, *p* < 0.001; nest height—*F*_(2, 64)_ = 10.6, *p* = 0.002] as in general, female mice tended to make smaller, less dense nests compared to males.

To finally test if deletion of Nrxn2α had a general effect on cognition, the Morris water maze was used to assess spatial memory (Figures 3C–F). All mice showed a significant reduction in latency [session factor—*F*_(7, 448)_ = 31.1, *p* < 0.001] and path length [session factor—*F*_(7, 448)_ = 14.1, *p* < 0.001] to the platform, indicating that spatial learning had occurred across the genotypes, with no significant sex effects [latency—*F*_(7, 448)_ = 1.85, *p* > 0.05; path length—*F*_(7, 448)_ = 1.84, *p* > 0.05]. Although there was a significant sex x genotype effect [*F*_(2, 64)_ = 6.41, *p* = 0.003] on swim speed, this was due to a trend for a decrease [*F*_(2, 32)_ = 3.98, *p* = 0.06] in male KO but an increase [*F*_(2, 32)_ = 0.39, *p* = 0.67] in female KO mice. During the probe task of the Morris water maze, all mice spent significantly more than 25% of the time in the target quadrant, further demonstrating that all mice had learnt the location of the platform and that there were no impairments in spatial memory in the Nrxn2α KO mice. In addition, working/episodic-like memory was measured in the DMP task in the Morris water maze (Figures 3E,F). All mice displayed a significant “saving time” in their latency to reach the platform over the first four sessions [session factor—*F*_(3, 192)_ = 4.45, *p* = 0.004]. There was also a significant reduction in path length across each session [session factor—*F*_(3, 192)_ = 8.81, *p* < 0.001] but there were no effects of genotype on the DMP task.

### Combined deletion of Nrxn2α and Nrxn2β

**Figure 4:**
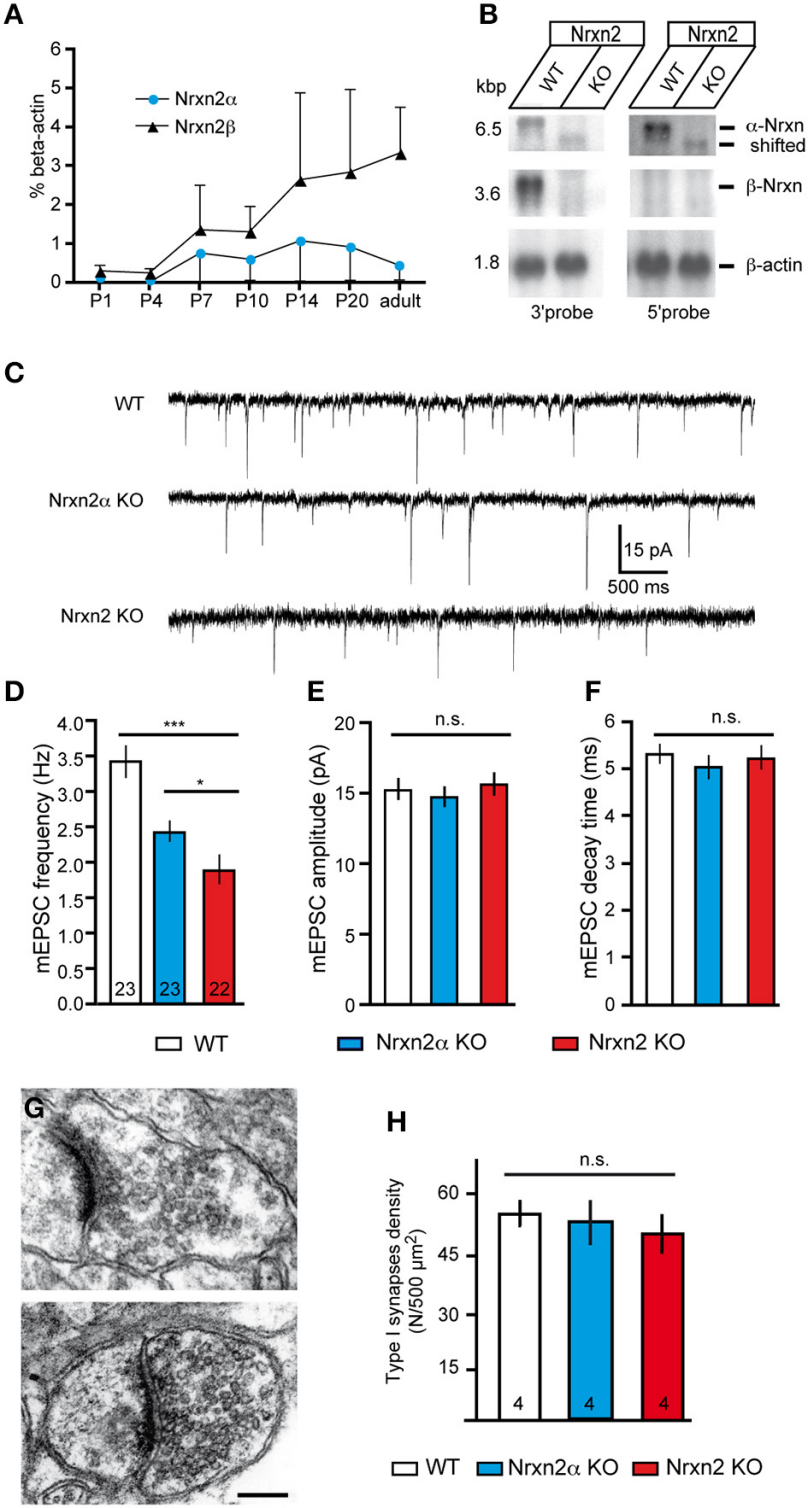
**Genetic targeting of *NRXN2* in mice alters excitatory neurotransmitter release**. **(A)** Expression of Nrxn2α and Nrxn2β mRNA during development was quantified by RT−PCR following postnatal day (P) 1, and normalized to β −actin levels. **(B)** Northern blots of total RNA from WT and Nrxn2 mutant (KO) mice hybridized with two riboprobes, using fragments from the 3′UTR that recognize Nrxn2α and Nrxn2β, and from the 5′UTR region that recognize only Nrxn2α. Re−hybridization against β −actin mRNA was used as loading control. **(C)** Representative current traces of miniature excitatory postsynaptic currents (mEPSC) recorded from layer V pyramidal cells in the primary somatosensory cortex of WT (upper trace), Nrxn2α KO (middle trace), and Nrxn2 KO mice (lower trace). **(D–F**) mEPSC frequencies **(D)** show a reduction for both variants of mutant mice but their amplitudes **(E)** and time constants such as decay times **(F)** did not differ. **(G–H)** Electron microscopic images of asymmetric, putative excitatory synapses of WT (**G**, upper panel), and Nrxn2 KO (**G**, lower panel) synapses from layer V of the somatosensory cortex, scale bar = 200 nm. Area densities of excitatory synapses (**F**, Gray type I) were averaged from all cortical layers of WT, Nrxn2α KO, and Nrxn2 KO mice. Data shown are means ± SEM (except in **A**, where they are SD). Numbers in bar graphs represent amount of recorded cells **(D–F)** or mice **(H)** per genotype. Statistical significances are indicated above the bars (^*^p < 0.05, ^***^p < 0.005, n.s. = not significant).

Nrxn transcripts are already present before birth (Puschel and Betz, 1995; Ullrich et al., 1995). To test if Nrxn2α and Nrxn2β are likely to contribute to neurodevelopmental disorders, we investigated their postnatal expression. Since no isoform-specific antibodies are available, we analyzed RNA levels extracted from whole brains by reverse-transcriptase real-time PCR (Figure 4A). Nrxn2α and Nrxn2β were both detectable at postnatal day 1 (P1), although expression levels were low. They rose prominently between P4-7, and increased until P14, coinciding with extensive synaptogenesis. While expression of Nrxn2β continued to rise until adulthood, Nrxn2α showed a decrease after P20 in mice (Figure 4A), reaching a similar difference as suggested by *in-situ* hybridization for rat neocortex (Ullrich et al., 1995). We chose the neocortex for our experiments because overall expression of Nrxn2 transcripts in this region is among the highest in brain, though 20–40% lower than Nrxn1 and Nrxn3 mRNAs (Aoto et al., 2013). Electrophysiological recordings were performed on animals between age P14-21 because in this period expression levels of α- and β-Nrxn2 are relatively high and cortical synapses mature.

Knockout mice of the complete NRXN2 gene were generated by targeting exon 23, which is shared by Nrxn2α and Nrxn2β (Tabuchi and Sudhof, 2002), and not spliced in 99.9% of full-length transcripts sequenced (Treutlein et al., 2014). Northern blots demonstrated that mRNA for Nrxn2β was undetectable in four independently processed KO brains, whereas for Nrxn2α a faint band shifted to lower molecular weight was visible (Figure 4B), presumably representing a small amount of truncated mRNA. Mice homozygous for the combined NRXN2 mutation (denoted as *Nrxn2 KO*) were derived in Mendelian ratio from heterozygous breedings, and WT littermates served as controls. Although Nrxn2 KO survived normally into adulthood, they had an about 25% lower body weight (WT 7.7 g ± 0.2, KO 5.8 g ± 0.2, *p* < 0.0001; 69 male/female WT and 51 KO at P16-21). Based on their expression profile, we compared P14-21-old mice of both models (Nrxn2α and Nrxn2) to controls in electrophysiological experiments.

### Impaired glutamatergic release in Nrxn2α and Nrxn2 mutants

Since mice lacking multiple α-Nrxn variants suffered from ubiquitously reduced vesicle release (Geppert et al., 1998; Missler et al., 2003; Kattenstroth et al., 2004; Zhang et al., 2005, 2010; Dudanova et al., 2006; Sons et al., 2006; Etherton et al., 2009), we performed whole-cell patch-clamp recordings from layer V pyramidal neurons of somatosensory cortex for our comparison of Nrxn2α and Nrxn2 mutants. In the neocortex, passive properties of layer V pyramidal cells such as input membrane resistance, resting potential and capacity were unchanged in Nrxn2 KO compared to control neurons (Table 1).

**Table 1:** **Neuronal properties and synaptic ultrastructure**.

	**WT**	**Nrxn2 KO**	***P***
**CELL PROPERTIES**			
Resting potential (mV)	−72.2 ± 0.6	−73.4 ± 0.8	0.256
Input resistance (MΩ)	100.4 ± 3.6	91.3 ± 4.1	0.099
Capacity (pF)	95.7 ± 5.2	110.8 ± 7.1	0.104
**ULTRASTRUCTURAL PARAMETERS**			
Length of synaptic zone (nm)	263.7 ± 16.8	276.9 ± 15.1	0.565
Width of synaptic cleft (nm)	13.2 ± 0.3	13.6 ± 0.3	0.623
Density of synaptic vesicles (N/μm^2^)	170.2 ± 13.1	197.6 ± 12.1	0.129
Area of synaptic terminals (μm^2^)	0.13 ± 0.0	0.15 ± 0.0	0.343

*Passive cell properties were analyzed at the beginning of each recording and quantitated for n = 40 cells in 10 animals from each genotype. Quantitative electron microscopy was performed on randomly chosen excitatory synapses for analysis of ultrastructural parameters, and quantitated for n = 35 terminals/ 3 WT animals, and n = 36 terminals/ 3 KO mice*.

To screen for defects in spontaneous transmission, we recorded excitatory miniature postsynaptic currents (mEPSCs) in presence of 1 μM TTX to abolish action potential driven network activity and 10 μM bicuculline to pharmacologically isolate excitatory (glutamatergic) events (Figure 4C). We observed that mEPSCs frequency is reduced to 65% of controls in young-adult Nrxn2α KO (WT: 3.42 ± 0.23 Hz, Nrxn2α KO: 2.43 ± 0.15 Hz, *p* < 0.001), similar to our analysis of brainstem synapses in newborn mutants (Missler et al., 2003), and to almost 50% in Nrxn2 KO mice (WT: 3.42 ± 0.23 Hz, Nrxn2 KO: 1.89 ± 0.21 Hz, *p* < 0.0001) (Figure 4D). The small difference between Nrxn2α and Nrxn2 KO neurons (Figure 4D) suggests that the combined loss of Nrxn2α and Nrxn2β only marginally aggravates the release phenotype. We also analyzed amplitudes and decay times of mEPSCs but found no significant changes (Figures 4E,F), indicating that transmitter quanta and receptors properties are not grossly altered in spontaneous transmission.

### Normal excitatory synapse formation in absence of Nrxn2

Nrxn and their binding partners were shown to induce synaptic contacts when overexpressed in cultures, experiments that proved particularly effective with β-Nrxn variants (Scheiffele et al., 2000; Graf et al., 2004; Nam and Chen, 2005; Ko et al., 2009). Conversely, knockdown with siRNA demonstrated synapse reduction *in vitro* (Chih et al., 2006; De Wit et al., 2009; Ko et al., 2011; Shipman et al., 2011). Thus, to evaluate if the reduced mEPSC frequency was due to altered synapse density in brain tissue, we performed quantitative electron microscopy (Figures 4G,H). In the somatosensory cortex, the number of asymmetric, presumably excitatory synapses (Figure 4G) was determined as an area density over all cortical layers but did not differ significantly between WT and both mutants (Figure 4H), confirming earlier data from the visual cortex of α-Nrxn double KOs (Dudanova et al., 2007). To exclude more subtle effects on ultrastructure of excitatory contacts, we also measured the area density of synaptic vesicles, length of synaptic zones, width of synaptic clefts and the area of synaptic terminals. No differences were found in mice lacking Nrxn2 compared to WT littermates (Table 1), indicating that even the combined deletion of Nrxn2α and Nrxn2β did not impair important morphological properties. These data suggest that differences observed in spontaneous excitatory transmission (Figure 4D) were not caused by structural defects, consistent with earlier conclusions (Missler et al., 2003; Varoqueaux et al., 2006; Dudanova et al., 2007).

### Investigation of short-term plasticity

**Figure 5:**
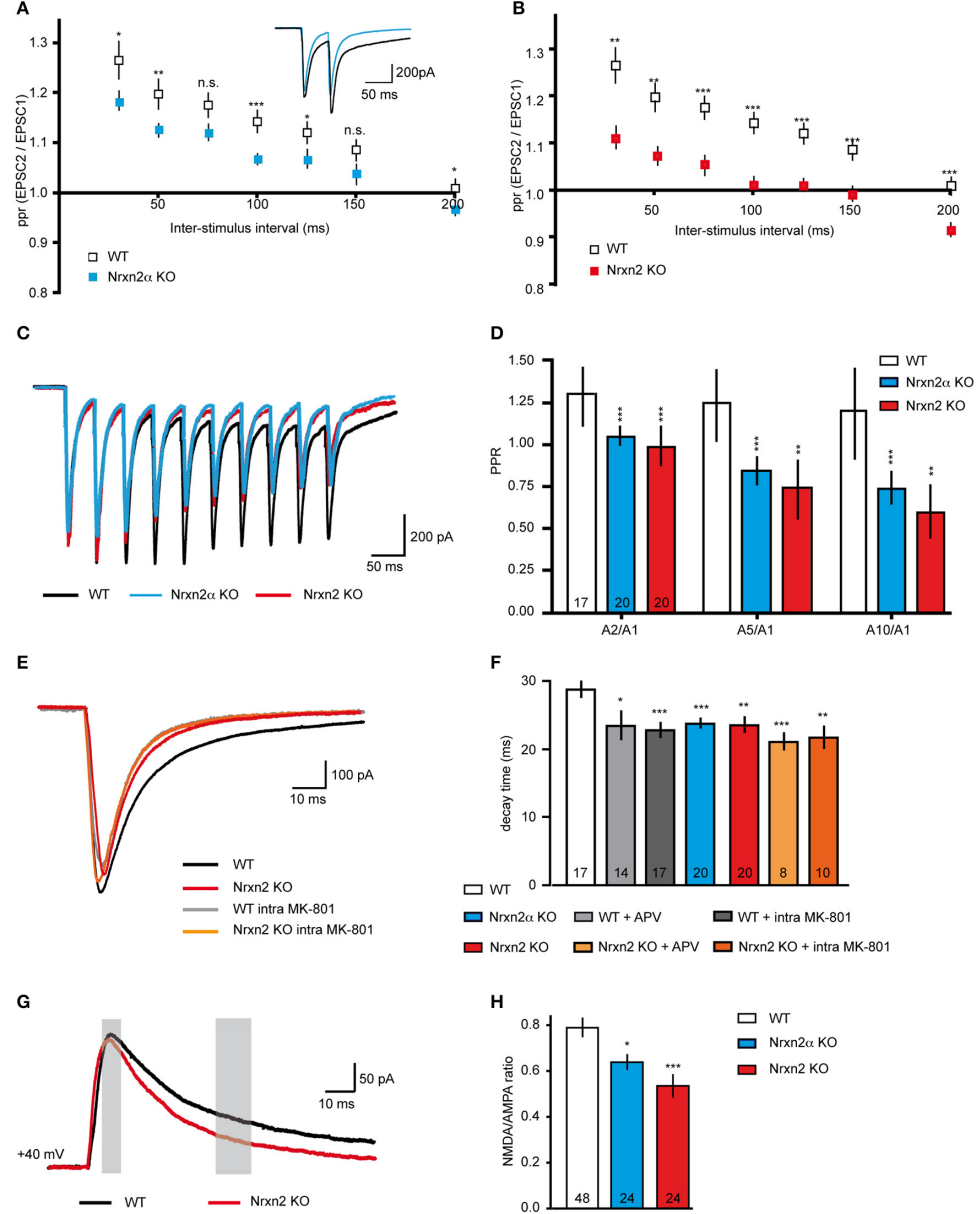
**Altered short-term plasticity and NMDAR function in Nrxn2 mouse models**. **(A,B)** Paired−pulse ratio (ppr) analysis of excitatory synapses comparing facilitation at different inter−stimulus intervals in WT to Nrxn2α **(A)** and to Nrxn2 **(B)** KO neurons. Inset in **(A)** represents averaged current samples from five individual traces, and numbers of recorded cells were *n* = 30 (WT), *n* = 34 (Nrxn2α KO), and *n* = 21 (Nrxn2 KO), from at least five mice per condition. **(C)** Averaged current traces of 20 Hz stimulus trains (10 pulses) recorded from cortical neurons of WT and both Nrxn2 mutant mouse models. **(D)** Analysis of second to first (A2/A1), fifth to first (A5/A1) and tenth to first (A10/1) stimuli ratios. Only WT neurons responded with robust facilitation. **(E)** Averaged current traces of evoked excitatory postsynaptic currents (eEPSCs) in WT (black trace, control; gray, with postsynaptically applied NMDAR blocker MK-801), and Nrxn2 KO mice (red, control; orange, with MK-801). **(F)** Monoexponential analysis of the deactivation kinetics (decay time) at half maximum stimulation strengths reveals reduction for both mutants. Pharmacological blockade of NMDAR with extracellularly applied APV and intracellular MK-801 mimicked this reduction in WT neurons (gray shaded bars) but had no further effect in KO (orange shaded bars). **(G)** Averaged current traces for analysis of NMDA/AMPA ratios recorded from WT, Nrxn2α KO, and Nrxn2 KO neurons. Responses of two components of ESPCs, mostly AMPAR-mediated (thin gray bar, 10 ms after stimulus onset) and mostly NMDAR-mediated (thick gray bar, after 40 ms) were isolated. **(H)** Quantitative ratios show a reduced NMDAR-mediated component in KO. Numbers in bar graphs **(D,F,H)** represent recorded cells per genotype, derived from at least five animals per condition. Data shown are means ± SEM. Statistical significances are indicated above the bars (^*^p < 0.05, ^**^p < 0.01, ^***^p < 0.005, n.s. = not significant).

Since reduced mEPSC frequencies might be caused by presynaptic mechanisms (Engelman and MacDermott, 2004), we also explored paradigms of short-term plasticity (Zucker and Regehr, 2002; Abbott and Regehr, 2004; Blitz et al., 2004). We applied paired-pulse stimuli to evoke plasticity at excitatory synapses, and calculated paired-pulse ratios (ppr). The stimuli reliably induced robust paired-pulse facilitation (PPF) in WT neurons as expected (Debanne et al., 1996; Frick et al., 2007; Feldmeyer and Radnikow, 2009), and depended in size on the 20–200 ms inter-stimulus intervals (Figures 5A,B). PPF was significantly reduced at most intervals in Nrxn2α–deficient neurons (Figure 5A), and almost abolished in Nrxn2 KO (Figure 5B), suggesting that additional removal of Nrxn2β again only modestly aggravates the phenotype.

To test short-term plasticity with another protocol, we applied 20 Hz stimulus trains (Myme et al., 2003; Li et al., 2005). Stimulation with 10 pulses at an inter-stimulus interval of 50 ms (repeated 5x with 20 s delay) induced robust facilitation at WT excitatory synapses over the whole train, but not at Nrxn2α and Nrxn2 KO terminals (Figure 5C). In WT neurons, the ratio of the A2/A1 stimuli was comparable to ratios of A5/A1 or A10/A1 (Figure 5D). Cells from both mutant strains, in contrast, showed almost no facilitation or even mild depression during stimulus trains (Figure 5D), consistent with the paired-pulse protocol (Figures 5A,B). These results may indicate that Nrxn2 is involved in normal expression of short-term plasticity at excitatory cortical synapses, or that additional defects exist that lead to such a reduction of facilitation. The observed reduction of PPF in both KO mouse lines, however, was not due to the summation of increased NMDAR-mediated EPSC tails (compare Altered NMDAR Function) over successive stimulations (data not shown).

### Altered NMDAR function

Short-term plasticity is frequently regarded as a presynaptic event based on residual Ca^2+^ (Zucker and Regehr, 2002; Abbott and Regehr, 2004) but additional components, in particular NMDAR, may also play a role (Carroll and Zukin, 2002; Malinow and Malenka, 2002; Lau and Zukin, 2007; Chamberlain et al., 2008). To test this possibility, we repeated the paired-pulse experiments in presence of the NMDAR antagonist APV. Facilitation was induced at cortical excitatory neurons from WT, however, PPF was abolished when we washed-in APV. Quantification of ppr at increasing inter-stimulus intervals showed that APV reduced WT values to the level of Nrxn2 KO, whereas application of the blocker during paired-pulse stimuli in KO did not change their responses (e.g., ppr at 100 ms interval, WT 1.30 ± 0.05, *n* = 25 cells, WT+APV, 1.06 ± 0.02, *n* = 14 cells, *p* = 0.0003; Nrxn2 KO 1.06 ± 0.02, *n* = 11 cells, Nrxn2 KO+APV, 1.05 ± 0.02, *n* = 14, *p* = 0.71).

To directly explore if NMDAR function was affected in Nrxn2α and Nrxn2 KO, we assessed the NMDAR-mediated component of evoked excitatory transmission (Figures 5E–H). Since glutamatergic transmission is mediated by both non-NMDA (AMPA and kainate receptors) and NMDA receptors, evoked EPSCs consist of their combined activation (Monaghan et al., 1989; Rivadulla et al., 2001). Using single electrical stimulations at half maximum strength (80–100 μA), we analyzed peak amplitudes and decay time constants of evoked EPSCs (Figures 5E,F). EPSCs amplitudes were moderately reduced in Nrxn2 KO neurons (WT: −875.20 ± 69.33 pA, *n* = 25 cells; KO: −543.60 ± 71.49 pA, *n* = 19 cells, *p* = 0.002*)*, consistent with earlier observations at brainstem synapses in Nrxn2α KO (Missler et al., 2003; Zhang et al., 2005). More interestingly, however, the deactivation kinetics of eEPSCs, a critical parameter in the control of synaptic integration (Paoletti et al., 2013), were changed in KO neurons toward faster decay (Figure 5E). Importantly, the effect of the Nrxn2α and Nrxn2 mutations on decay time could be mimicked by washing-in the NMDAR antagonist APV during recordings from WT neurons, or by adding MK-801 into the pipette solution (Figure 5E), an NMDAR blocker suitable for intracellular application. Application of APV or MK-801 to KO neurons (Figure 5E), in contrast, did not further change responses. Bath application of APV and intracellular addition of MK-801 had a similar effect on decay times of eEPSCs recorded from WT cells but failed to alter the reduced decay times of Nrxn2α and Nrxn2 KO (Figure 5F). These results suggest that mostly postsynaptic NMDAR populations may be involved in the deactivation kinetics phenotype.

To validate the effect on NMDAR, we determined the ratio of NMDAR and AMPAR-mediated postsynaptic currents (Myme et al., 2003; Jung et al., 2010). NMDA/AMPA ratios were calculated by applying single electrical stimuli first at −80 mV to define the AMPAR-mediated peak which occurred at 10 ms after stimulation artifact (left gray bar, Figure 5G). Cells were then depolarized stepwise up to +40 mV and the NMDAR-mediated component calculated 40 ms after the stimulus (right gray bar, Figure 5G) (Myme et al., 2003). These recordings were performed in presence of 2 μM bicuculline to block IPSCs. Quantitative analysis of NMDA/AMPA ratios revealed an almost 25% reduction in Nrxn2α KO and a 35% reduction in Nrxn2 KO neurons compared to WT (Figure 5H). The data indicate that NMDAR-mediated transmission is moderately altered even in absence of a single Nrxn variant. While our current findings are consistent with the diminished NMDA/AMPA ratio of eEPSCs found in cultured neocortical slices from KO mice lacking all α-Nrxn (Kattenstroth et al., 2004), there are also important differences (see Discussion).

### Neocortical GABAergic transmission is unchanged in Nrxn2α and Nrxn2 mutants

Since earlier analysis showed defects of inhibitory transmission in multiple α-Nrxn KO (Missler et al., 2003), we also investigated inhibitory synapses in Nrxn2 mutants. Although a small reduction of GABAergic mini frequency was present in brainstem neurons of newborn Nrxn2α KO (Missler et al., 2003), the mIPSC frequency remained unchanged in the cortical neurons of young-adult KO studied here (WT, 1.61 ± 0.14 Hz, *n* = 14 cells/8 mice; Nrxn2 KO, 1.51 ± 0.15 Hz, *n* = 9 cells/4 mice, *p* = 0.657). Consistent with the earlier study, no differences were seen for mIPSC amplitudes (WT, 7.82 ± 0.43 pA, *n* = 14 cells/8 mice; Nrxn2 KO, 8.43 ± 0.24 pA, *n* = 9 cells/4 mice, *p* = 0.27) and decay times (WT, 9.76 ± 0.96 ms, *n* = 14 cells/8 mice; Nrxn2 KO, 9.60 ± 0.68 ms, *n* = 9 cells/4 mice, *p* = 0.29). In accordance, the density of symmetric, presumably inhibitory, synapses in the somatosensory cortex were unchanged in Nrxn2 KOs compared to littermates (WT, 5.5 ± 2.1/100 μm^2^, *n* = 4 mice; Nrxn2 KO, 5.8 ± 1.7/100 μm^2^, *n* = 4 mice; *p* = 0.93). Similarly, no differences in short-term plasticity as assessed by 20 Hz stimulus trains (data not shown) or by paired-pulse stimuli were observed when we compared inhibitory cortical synapses from Nrxn2 KO mice with controls. At short inter-stimulus intervals (20–30 ms), both genotypes expressed moderate facilitation (ppr 20 ms: WT, 1.13 ± 0.04, *n* = 10 cells; Nrxn2 KO, 1.18 ± 0.05, *n* = 12 cells, *p* = 0.40). At longer inter-stimulus intervals (75–200 ms), inhibitory WT as well as Nrxn2 KO synapses showed properties of a paired-pulse depression (ppr 150 ms: WT, 0.90 ± 0.02, *n* = 10 cells; Nrxn2 KO, 0.89 ± 0.02, *n* = 13 cells, *p* = 0.85), as expected for these neurons (Fleidervish and Gutnick, 1995; Reyes et al., 1998). Together, our results indicate that constitutive deletions of the Nrxn2 gene predominantly affect glutamatergic release in the neocortex after postnatal maturation, supporting the emerging picture that region- and/or age-specific susceptibilities exist as suggested before for other mutations in Nrxn (Etherton et al., 2009) or Nlgn (Gibson et al., 2009; Blundell et al., 2010; Etherton et al., 2011b; Soler-Llavina et al., 2011).

## Discussion

Previous studies in genetically modified mice dissected the function of Nrxns either in single Nrxn1α KO (Geppert et al., 1998; Etherton et al., 2009; Laarakker et al., 2012; Grayton et al., 2013), a splice variant-specific Nrxn3 KO (Aoto et al., 2013), or in multiple α−Nrxn KO mice (Missler et al., 2003; Kattenstroth et al., 2004; Zhang et al., 2005; Dudanova et al., 2006, 2007; Sons et al., 2006). Very little information is available on Nrxn2α KO, which initially served as the least affected “KO control” for multiple α−Nrxn KO (Missler et al., 2003; Zhang et al., 2005). In addition, no report exists on simultaneous inactivation of α− and β −variants of any NRXN gene. We chose Nrxn2 for such a combined deletion because recent work has identified novel mutations in ASD patients in this gene (Gauthier et al., 2011), extending into exons coding for its β −variant. In addition, Nrxn2 transcripts appear particularly prone to activity-dependent splicing (Rozic-Kotliroff and Zisapel, 2007; Rozic et al., 2013), and the strong expression of its β-variant during postnatal development suggested that any clear modification of the Nrxn2α KO phenotype should be predominantly due to the lack of Nrxn2β. Here, we tested both mouse models, previously generated single Nrxn2α (Missler et al., 2003) and the combined Nrxn2 KO, for functional defects in neurotransmission and synapse formation. However, we had to restrict behavioral testing to the Nrxn2α model already backcrossed into a pure C57bl6 background to avoid confounding effects from mixed genetic backgrounds (Bucan and Abel, 2002; Wolfer et al., 2002) that made recent behavioral studies of Nrxn1α KO mice difficult to compare (Etherton et al., 2009; Laarakker et al., 2012; Grayton et al., 2013).

The behavioral profile of Nrxn2α KO mice revealed in this study differs from that reported for Nrxn1α KO (Etherton et al., 2009; Laarakker et al., 2012; Grayton et al., 2013). Nrxn1α KO mice backcrossed onto the identical genetic background as Nrxn2α KO display a decrease in social investigation, an increase in aggression, an impairment in nest building, and reduced locomotor activity in novel environment (Grayton et al., 2013). In comparison, we found that homozygous deletion of the Nrxn2α gene led to reduced sociability, decreased social investigation, and increased grooming behaviors only in the female mice. Furthermore, both male and female Nrxn2α KO mice displayed increased anxiety in the light-dark box and elevated plus maze, and impairments in nest-building behaviors; however they did not display any deficits in spatial learning or working/episodic-like memory. These results suggest that deletion of Nrxn2α leads to alterations in social and repetitive behaviors in a sex specific manner, but do not affect cognition. Both of these behaviors are within core symptom domains affected in ASDs, as well as general heightened anxiety, which manifests in some patients with neurodevelopmental disorders (Cosoff and Hafner, 1998; White et al., 2009). Recently, the behavior of Nrxn2α homozygote mice has been examined on an isogenic background, however heterozygote mice and sex differences were not investigated (Dachtler et al., 2014). Here, the Nrxn2α KO mice failed to show any significant preference for sociability or social novelty, and displayed an anxiety phenotype in the open field and elevated plus maze tasks. These results are similar to those shown here, where we see that homozygote deletion of Nrxn2α lead to alterations in social behaviors and an increase in anxiety. However, reduced sociability and decreased social investigation only was found in female KO mice, stressing the importance of investigating sex differences in behavioral studies. Furthermore, the current study has included both heterozygote and KO mice, which allows the behavior of the Nrxn2α model to be further explored. Currently there is no clear causal role for the loss of Nrxn2α in psychiatric disorders, due to only a few mutations in patients having been found (Gauthier et al., 2011; Mohrmann et al., 2011). However, examining the effects of both the heterozygote and homozygote deletion will contribute to understanding any association between this gene and behavioral alterations associated with psychiatric disorders such as ASD.

The functional differences between the three α-Nrxn isoforms are not yet understood, but previous research has provided some insight. Transgenic overexpression of a single Nrxn1α variant can reverse the synaptic transmission phenotype in KO mice, independent of how many α-Nrxns were deleted (Zhang et al., 2005). This suggests functional redundancy between isoforms, and that the α-Nrxns do not exhibit fundamental mechanistic differences. Thus, a hypothesis to explain the phenotypic difference seen in Nrxn1α and Nrxn2α KO mice is that the isoforms have a different spectrum of binding partners, which may differ in various brain regions or synaptic subpopulations. For example, alternative splicing at spice site #4 was shown to cause differential interactions with several ligands (Ichtchenko et al., 1995, 1996; Boucard et al., 2005; Ko et al., 2009; Siddiqui et al., 2010; Uemura et al., 2010), and splice variants in Nrxn1 and Nrxn2 are in fact differentially distributed in brain regions (Ullrich et al., 1995; Aoto et al., 2013). In addition, other target molecules of α-Nrxn function such as GABA_A_R (Zhang et al., 2010), GABA_B_R (Dudanova et al., 2006; Fu and Huang, 2010), AMPAR (Aoto et al., 2013), NMDAR (Kattenstroth et al., 2004), or voltage-gated Ca^2+^ channels (Missler et al., 2003; Zhang et al., 2005) have yet to be studied for preferences by different α-Nrxn isoforms. With regards to behaviors of Nrxn1α (Etherton et al., 2009; Laarakker et al., 2012; Grayton et al., 2013) and Nrxn2α KO (studied here, and by Dachtler et al., 2014), there is not enough information available to assign behavioral differences to functional differences in the isoforms.

The loss of Nrxn2β in our new combined Nrxn2 mutant is unequivocally demonstrated by absence of its mRNA, however, some truncated Nrxn2α may still be present, possibly caused by unexpected splicing events around exon 23 (Tabuchi and Sudhof, 2002; Treutlein et al., 2014). Although adverse effects of modified mRNA species can never be ruled out, it appears unlikely that the deletion translates into a functional Nrxn2α because it could not be anchored in the presynaptic membrane for lack of a transmembrane region (Ushkaryov et al., 1992; Ushkaryov and Sudhof, 1993). Moreover, dominant-negative effects of a potentially secreted Nrxn2α variant also appear unlikely because the possibility of such isoforms does exist in brain, at least in the closely homologous Nrxn3α (Ushkaryov and Sudhof, 1993; Aoto et al., 2013; Treutlein et al., 2014).

We show here that deletions of Nrxn2α and the combined Nrxn2 cause similar impairments of mEPSC frequency at neocortical synapses but no change in their density. These results are in line with our earlier analysis of multiple α-Nrxn KO mice, albeit the reduction in mEPSC frequency was stronger in double or triple KOs (Missler et al., 2003; Zhang et al., 2005). The similarly prominent reduction in GABAergic frequency identified in brainstem synapses of newborn mutants (Missler et al., 2003; Zhang et al., 2005) was not present in our current analysis in cortical neurons of young-adult mutants. This is consistent with the study of single Nrxn1α KO that also found mEPSC, but not mIPSC, frequencies reduced in the CA1 region of hippocampus (Etherton et al., 2009). Interestingly, we observed little differences between Nrxn2α and Nrxn2 KOs in mEPSC, evoked EPSC and PPF deficiencies, despite the high expression levels of Nrxn2β in most neocortical areas (Ullrich et al., 1995; Aoto et al., 2013). Our comparison of Nrxn2α and Nrxn2 KO mice, the latter with additional deletion of Nrxn2β, suggests that the function of evolutionarily older α-Nrxns (Tabuchi and Sudhof, 2002; Haklai-Topper et al., 2011) is pre-eminent, or that no role exists for Nrxn2β at excitatory synapses in the somatosensory cortex. This scenario is consistent with rescue experiments which revealed that the triple α-Nrxn KO phenotype can be compensated by transgenic overexpression of Nrxn1α but not by Nrxn1β (Missler et al., 2003; Zhang et al., 2005). To exclude that a high redundancy among different β-Nrxn obscured important functions in our single gene deletion, future work will have to address their contribution systematically.

A role of Nrxn2 in short-term plasticity was not investigated before, but transfection of a dominant-negative Nrxn1 enhanced PPF in hippocampal slice cultures (Futai et al., 2007). The reduced PPF at glutamatergic synapses observed here is consistent with that study. The finding is also surprising because an inverse relationship between the degree of facilitation and release probability (P_r_) is frequently observed (Debanne et al., 1996; Dobrunz and Stevens, 1997; Murthy et al., 1997) but not present here. Thus, explanation of paired-pulse and frequency-dependent plasticity solely by initial release probability is too simplistic: for example, Rab3A KO showed enhanced PPF in absence of reduced P_r_ (Geppert et al., 1997), and synapsin1/2 KO exhibited enhanced depression without change in P_r_ (Rosahl et al., 1995; Sun et al., 2006). At present, we cannot decide definitively if the reduced facilitation unveiled here is caused by presynaptic mechanisms alone (e.g., residual calcium) or by a combination of pre- and postsynaptic defects, quite possible for a molecule as Nrxn2 which engages in trans-synaptic complexes (Reissner et al., 2013). In support of a combined effect, involving altered NMDAR function, we found that application of NMDAR antagonists abolished the differences between WT and Nrxn2α or Nrxn2 KO. In addition, decay times of the EPSCs at Nrxn2 KO synapses are faster and accumulation of EPSCs at shorter inter-stimulus intervals in ppr experiments is less pronounced. These findings provide evidence that NMDAR are involved in the facilitation phenotype because the slow component of the EPSCs are reduced in KO mice, and this could be mimicked in WT neurons by pharmacologically blocking postsynaptic NMDARs. In agreement of such a possibility, it has previously been reported that postsynaptically located NMDAR interact with L-type calcium channels, and are able to modify paired-pulse potentiation (Zinebi et al., 2001; Akopian and Walsh, 2002).

The NMDA/AMPA ratios found altered here in Nrxn2α or Nrxn2 KO are consistent with our earlier study of α-Nrxn triple mutants that displayed an about 50% reduction of NMDA/AMPA ratio in cultures (Kattenstroth et al., 2004). One discrepancy with this study remains, however, as the effect on NMDAR was earlier determined as cell-autonomous: co-culturing GFP-expressing WT neurons on Nrxn2α (control) and α-Nrxn triple KO slices failed to reveal a changed NMDA/AMPA ratio (Kattenstroth et al., 2004). While this discrepancy cannot be completely resolved, the earlier study relied solely on NMDA/AMPA ratios of mini events under Mg^2+^-free conditions (Kattenstroth et al., 2004), while our investigation here is based on robust NMDAR-mediated components of evoked EPSCs in acute slices at high stimulus intensities. This notwithstanding, our current data in Nrxn2α and Nrxn2 KO agree with many studies that reported dysfunction of NMDAR alters synaptic plasticity (Malinow and Malenka, 2002; Zucker and Regehr, 2002; Li et al., 2009; Jung et al., 2010), and is associated with psychiatric disorders (Lau and Zukin, 2007; Li et al., 2009).

Our observation of impaired postsynaptic NMDAR function emphasizes the importance of signaling through the association of Nrxn with *trans*-synaptic binding partners (Reissner et al., 2013). Their interaction partner Nlgn (Ichtchenko et al., 1995; Boucard et al., 2005) was actually shown to affect NMDAR recruitment via postsynaptic scaffold proteins (Irie et al., 1997; Song et al., 1999; Barrow et al., 2009). Consequently, other studies demonstrated that NMDAR depend directly on Nlgn because NMDAR-mediated short- and long-term plasticity is altered when their interaction is disrupted (Blundell et al., 2010; Jung et al., 2010; Etherton et al., 2011a; Budreck et al., 2013). In fact, dysfunction of behaviors as reduced social novelty seeking, often associated with autism, can be induced in mice by lowering levels of the NMDAR (Mohn et al., 1999; Finlay et al., 2015). Such investigations may help to explain that the behavioral phenotype in Nrxn2α KO mice is related to the changes in short-term plasticity and altered NMDAR function. More research is needed, however, to determine how the Nrxn/Nlgn complex regulates the function of NMDAR at the cellular level, and to narrow the gap between molecular manipulations and altered behaviors.

## Author contributions

All authors gave their approval of the manuscript and agree to take responsibility for the integrity of their data and the accuracy of their data analysis. Study concept and experimental design was done by MM and CF. Acquisition of data and analysis were performed by GB, HMG, AR, HL, ID and BWW. Interpretation of data was done by GB, HMG, AR, DAC, CF and MM. The article was drafted by MM and critically revised by all authors.

### Conflict of interest statement

The authors declare that the research was conducted in the absence of any commercial or financial relationships that could be construed as a potential conflict of interest. Regarding the financial disclosure, the authors confirm that David A. Collier and Hannah Grayton are full time employees of Eli Lilly and Company Ltd. David A. Collier is also a stockholder of Eli Lilly and Company, and a visiting professor at the Institute of Psychiatry. Eli Lilly and company provided an unrestricted educational grant to contribute to the breeding costs of the NRXN2α mice used in the present study. The present study does not relate to any patents, intellectual property, products in development or marketed products by Eli Lilly and Company. Cathy Fernandes has received an honorarium from Eli Lilly and Company Ltd. as a seminar speaker. This does not alter the authors' adherence to all of Frontier's policies on sharing data and materials.
